# A New Approach for Using Genome Scans to Detect Recent Positive Selection in the Human Genome 

**DOI:** 10.1371/journal.pbio.0050171

**Published:** 2007-06-19

**Authors:** Kun Tang, Kevin R Thornton, Mark Stoneking

**Affiliations:** 1 Department of Evolutionary Genetics, Max Planck Institute for Evolutionary Anthropology, Leipzig, Germany; 2 Department of Ecology and Evolutionary Biology, University of California Irvine, Irvine, California, United States of America; University of Dublin, Ireland

## Abstract

Genome-wide scanning for signals of recent positive selection is essential for a comprehensive and systematic understanding of human adaptation. Here, we present a genomic survey of recent local selective sweeps, especially aimed at those nearly or recently completed. A novel approach was developed for such signals, based on contrasting the extended haplotype homozygosity (EHH) profiles between populations. We applied this method to the genome single nucleotide polymorphism (SNP) data of both the International HapMap Project and Perlegen Sciences, and detected widespread signals of recent local selection across the genome, consisting of both complete and partial sweeps. A challenging problem of genomic scans of recent positive selection is to clearly distinguish selection from neutral effects, given the high sensitivity of the test statistics to departures from neutral demographic assumptions and the lack of a single, accurate neutral model of human history. We therefore developed a new procedure that is robust across a wide range of demographic and ascertainment models, one that indicates that certain portions of the genome clearly depart from neutrality. Simulations of positive selection showed that our tests have high power towards strong selection sweeps that have undergone fixation. Gene ontology analysis of the candidate regions revealed several new functional groups that might help explain some important interpopulation differences in phenotypic traits.

## Author Summary

The evolution of new functions and adaptation to new environments occurs by positive selection, whereby beneficial mutations increase in frequency and eventually become fixed in a population. Detecting such selection in humans is crucial for understanding the importance of past genetic adaptations and their role in contemporary common diseases. Methods have already been developed for detecting the signature of positive selection in large, genome-scale datasets (such as the “HapMap”). Positive selection is expected to more rapidly increase the frequency of an allele, and hence, the length of the haplotype (extent of DNA segment) associated with the selected allele, relative to those that are not under selection. Such methods compare haplotype lengths within a single population. Here, we introduce a new method that compares the lengths of haplotypes associated with the same allele in different populations. We demonstrate that our method has greater power to detect selective sweeps that are fixed or nearly so, and we construct a statistical framework that shows that our method reliably detects positive selection. We applied our method to the HapMap data and identified approximately 500 candidate regions in the human genome that show a signature of recent positive selection. Further targeted studies of these regions should reveal important genetic adaptations in our past.

## Abstract 3

The authors present a novel genome-scan approach for detecting local positive selection. The approach is designed to detect selective events that have resulted in complete or near-complete fixation of a beneficial allele.

## Introduction

In approximately the past 50,000 years, anatomically modern human emerged from Africa and colonized most of the globe. During this relatively short time, humans encountered numerous novel environments with drastically different climates, pathogens, and food sources. In addition, several important cultural developments undoubtedly had far-reaching consequences, such as the introduction of various forms of agriculture, the domestication of animals, and increasing population density, resulting in conditions favorable for epidemics and infectious diseases. It thus seems likely that there has been ample opportunity for human populations to have adapted by natural selection to the extreme changes that have accompanied their recent expansion.

Detection of recent, local positive selection has long been a pivotal issue of population genetics and is of increasing interest to human geneticists [1–7]. However, demonstrating conclusively that local selection has operated on a gene remains a difficult task because it involves several aspects: demonstrating that patterns of allelic variation at the gene are not consistent with neutrality; that there is a functional difference between alleles; and finally, that the functional difference would result in a phenotypic effect that would be influenced by selection. Such efforts have been mostly focused on individual candidate genes [6], and very few have had all these aspects demonstrated to some degree at least; examples of genes for which all these aspects have been shown include the lactose tolerance gene *LCT* [8], salt-regulation genes at the *CYP3A* cluster [9], several disease resistance genes, e.g., *G6PD* and *CASP12* [10,11], and pigmentation genes, e.g., *SLC24A5* and *MATP* [12,13].

Recently, high-density surveys of genetic variation across the genome are available for several major human populations [14,15]; and the advent of genotyping technology makes such whole-genome surveys increasingly feasible in any specific group [16,17]. This allows systematic characterization of recent positive selection in the human genome, and several studies have recently used such datasets to identify regions of the human genome that harbor signatures of positive selection [18–23]. The obvious strategy, from a statistical standpoint, is to match the empirical data to a sensible neutral model and thereby detect significant departures from neutrality. However, this is complicated by the need for a neutral model that incorporates the demographic history of humans, which is unknown and hence arguably not practical, as well as the high sensitivity of conventional test statistics to the departures from neutral assumptions, such as bottlenecks and population structure [24]. Thus, aside from the composite-likelihood–based test [20], which shows good robustness towards several demographic factors, previous studies were largely restricted to identifying outlier loci from the genome-wide distribution of a certain test statistic, rather than defining clear cutoffs for distinguishing selection from instances of extreme neutral drift. This greatly constrained the use of genomic scans, because it is impossible to address essential questions such as the magnitude of recent positive selection across the genome and the false positive and false discovery rates of any particular approach.

Among the various statistics used for recognizing signals of positive selection from polymorphism data, the extended haplotype homozygosity (EHH), first introduced by Sabeti et al. [25] is particularly useful (e.g., [22,23,26–28]). EHH incorporates information on both allele frequency and the association between single nucleotide polymorphism (SNP) sites, and may provide higher testing power than conventional statistics [25]; furthermore, it is designed to work with SNP rather than sequencing data, being less sensitive to ascertainment bias than other approaches. These properties make EHH a very promising candidate strategy for genome-wide scans of recent positive selection. Voight et al. [21] recently introduced a powerful method for identifying alleles that have been driven to intermediate frequencies by positive selection, based on the comparison of EHH between alleles within a population, and detected wide-spread signals of positive selection in the human genome. However, this method lacks power to detect selective sweeps that have resulted in near or complete fixation of an allele in a population, and hence may fail to detect a significant fraction of loci that have experienced local positive selection.

Here, we describe a novel genome-scan approach for detecting local positive selection that is designed to detect selective events that have resulted in complete or near-complete fixation of a beneficial allele. Rather than comparing the EHH between alleles within one population, this approach contrasts the EHH patterns of the same allele between populations, analogous to other statistics (lnRV and lnRH [29,30]) that are also based on contrasting genetic diversity between populations. We introduce a simple counting algorithm to estimate EHH-related statistics directly from genotype data, which avoids the time-consuming, computationally intensive estimation of haplotypes. Moreover, we use simulations to demonstrate that our test has high power to detect fixed, strong selective sweeps, and that our new summary statistic is robust over a variety of demographic models of human history, while capturing apparent departures of the empirical data from neutrality. We apply our method to two genome-wide SNP datasets, define candidate regions for local positive selection, and identify functional gene categories that contain an excess of candidates.

## Results

### The Test Statistics

The primary goal of our study was to detect evidence of recent, local positive selection from the whole-genome SNP data of both the International HapMap Project and Perlegen Sciences [14,15]. For the Perlegen dataset, we used the data from all 71 unrelated individuals sampled in three groups: African American (23), European American (24), and Han Chinese (24). For the HapMap dataset, we only included unrelated individuals from three groups; specifically 60 Yorubans, 40 Europeans, and 45 Han Chinese (see Methods). Given the obvious shared ancestry between the groups in Perlegen and HapMap, we hereafter refer to them as Africans (Afr), Europeans (Eur), and Chinese (Chn), respectively.

Our approach is based on the idea of extended haplotype homozygosity (EHH). First proposed by Sabeti et al. [25], the EHH statistic is a measure of the decay of identity of haplotypes as a function of distance from a “core” allele, and the EHH associated with an allele that has risen to a particular frequency under neutrality is expected to differ from the EHH of an allele that has risen to the same frequency by positive selection. Under neutral genetic drift, a young derived allele that is at low frequency will have few associated recombination events, and therefore will have low haplotype diversity and high EHH, whereas a high-frequency ancestral allele will have high haplotype diversity and low EHH because of the many recombination events that have occurred. A young derived allele under positive selection, however, rises rapidly in frequency while retaining extensive EHH, and leaves the alternative allele in low frequency with low EHH.

Previous approaches compare the EHH decay between the alleles (hereafter, we refer to the EHH of an allele as EHHA) of a site/core-haplotype within a single population, so that the alleles with excessive EHH and high allele frequency indicate positive selection [21,25]. An obvious caveat of this approach is that the intrapopulation comparison has low power when the selected allele is at high frequency, and becomes impossible when the selected allele is fixed. Seeking a novel strategy to overcome this problem, our approach compares the decay of EHH of an individual SNP site (EHHS), rather than EHHA, between populations. EHHS is defined as the decay of identity of haplotypes starting from the tested SNP site of a population as a function of distance. Starting at site *i,* the normalized EHHS at site *j* would be:

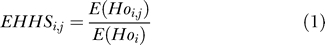



This is the haplotype homozygosity between *i* and *j* normalized by the homozygosity at site *i.* Note that both the haplotype homozygosity and homozygosity calculations are based on the site, regardless of the status of each allele.

**Figure 1:**
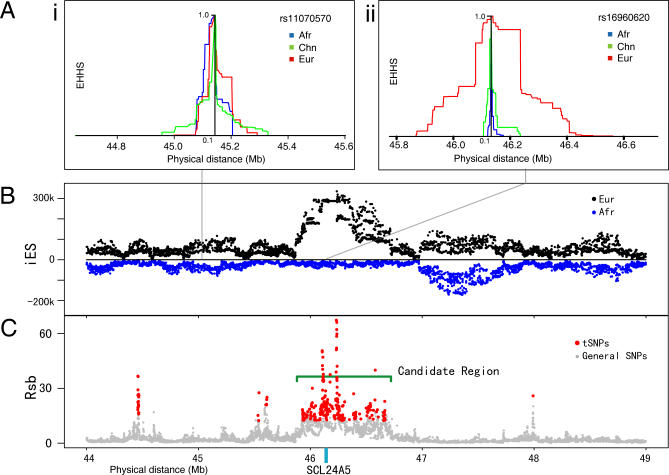
Signal of Positive Selection around the *SCL24A5* Gene in Europeans from the HapMap Data (A) The EHH decay plot for i, a neutral site outside the selective sweep; and ii, a site in the middle of the *SCL24A5* gene, which shows strong evidence of positive selection in Europeans (Eur). Afr, Africans; Chn, Chinese. (B) iES plotted against physical distance, back to back for Europeans and Africans. (C) Signals of positive selection shown in the plot of Rsb (European/African) against physical distance.

In principle, EHHS is roughly the average EHHA for the two alternative alleles weighted by their squared allele frequencies, and starts at a value of one and decays towards zero (Figure 1A). EHHS is therefore largely determined by the EHHA of the high-frequency allele, decaying very fast under neutrality when the dominating allele is the ancestral allele, or remaining extensive when the beneficial derived allele sweeps to a very high allele frequency or to fixation (Figure 1A).

When haplotype data are available, EHHS can be derived in an analytical way:

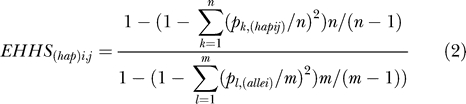



Where *P_k,_*_(*hap ij*)_ is the *k*th haplotype between *i* and *j,* and *P_l,_*_(*alle i*)_ is the *l*th allele (variant of a SNP site) for site *i.* However, inferring haplotypes from genotype data at a genome-wide scale is very computationally intensive and may not be accurate over long genomic distances [31–33]. Here, we propose a new way to estimate EHHS directly from the genotypic data. Assuming random mating in a population, each extant individual represents an instance of random chromosome (haplotype) pairing; the proportion of homozygotes in a population, therefore, serves as an estimator of the homozygosity. The EHH can then simply be estimated as the proportion of individuals that remain homozygous for intervals starting from the tested SNP and extending in both directions.

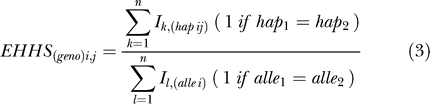



Here the *I_k,_*_(*hap ij*)_ is the identity of the two haplotypes between site *i* and *j* in one individual, and *I_l,_*_(*alle i*)_ is the identity of the alleles at site *i.*

Because this procedure is based on counting the number of homozygotes, hereafter, we call it the counting algorithm. Our major analyses, including the simulations and the analysis of the empirical HapMap and Perlegen genotype data, use this procedure. Treatment of missing data is discussed in the Methods section. This algorithm is fast and not computationally intensive. Although there is some inevitable loss of information compared to analyses based on phase-known data, we show later that the analysis based on the counting algorithm is comparable to that based on phase-known data.

We use the integrated EHHS (iES) to summarize the EHHS decay for one single site, which basically integrates the area under the curve of EHHS against distance (see Methods; Figure 1A). The log ratio of iES values between population (ln(Rsb)′) then compares the EHHS decay of a single site between two populations:

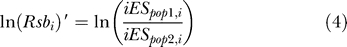



Because recombination rates are largely conserved among human populations [34], the comparison between populations thereby provides an internal control that largely cancels out the effect of heterogeneous recombination rates. Extreme values of ln(Rsb)′ indicate much slower EHH decay in one population than the other, and therefore represent possible evidence of selection. To achieve robustness across different demographic scenarios, ln(Rsb)′ is standard transformed as:

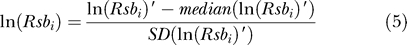



Here *SD* refers to standard deviation; the median is used instead of the mean because it is less sensitive to extreme data points. We calculated ln(Rsb) for every SNP site in the HapMap phase II and Perlegen data, and for each pairwise comparison among the three populations, namely African versus European (AE), African versus Chinese (AC), and Chinese versus European (CE).

We compared the ln(iES) and ln(Rsb)′ distributions estimated from the counting algorithm and from the analytical calculation based on a phase-known dataset, the phase I HapMap data (see Methods). The agreement between the two methods is considerable; *r* ^2^ ranged from 0.855 ~ 0.914 across different comparisons for ln(iES) and from 0.647 ~ 0.731 for ln(Rsb). Higher SNP densities, such as in the full Perlegen and HapMap datasets, should give even better agreement.

### Effects of Recombination Rate and SNP Density

In principle, our approach should be robust against varying local recombination rates and SNP density, because ln(Rsb) compares the relative iES of the same SNP between two populations, and such local effects are thus controlled. Nevertheless, we tested for any influence of recombination rate or SNP density by linear regression. Sites around large gaps in the SNP distribution were first excluded from further analysis because iES estimation is sensitive to such gaps (see Methods). We observed statistically significant, but low-level, associations between Rsb and either the recombination rate or the SNP density for all the comparisons (*r* ^2^ between ln(Rsb) and recombination rate is 0.0014–0.0086 for the HapMap data, and 0.0025–0.0153 for the Perlegen data; *r* ^2^ between ln(Rsb) and SNP density is 0.0002–0.0087 for the HapMap data, and 0.0004–0.02 for the Perlegen data, respectively). Given the low magnitude of these associations, we did not include any further corrections for recombination rate and/or SNP density.

### Comparison between HapMap and Perlegen Datasets

**Figure 2:**
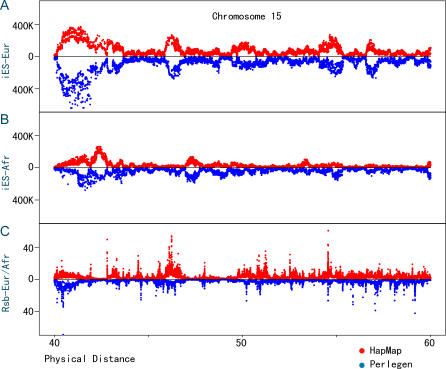
Comparisons of iES and Rsb Distributions between the Perlegen and HapMap Datasets The iES and Rsb values are plotted for a 20-Mb region on Chromosome 15. (A and B) iES against physical distance in Europeans (Eur) and Africans (Afr), respectively, back to back for the HapMap and Perlegen data. (C) Plot of Rsb of Europeans over Africans against physical distance, back to back for the HapMap and Perlegen data.

The Perlegen and HapMap datasets vary greatly in their SNP density, sample size, and ascertainment; it is, therefore, of interest to compare the iES and Rsb distributions between the two. We observed good agreement between the Perlegen and HapMap data for the iES distributions, especially for the comparisons in Europeans and Chinese (Figure 2A). Lower similarity in iES distributions is seen between the two African samples, which likely reflects admixture in the African Americans sampled by Perlegen (Figure 2B). The iES distribution is more variable in the Perlegen than in the HapMap dataset, probably due to the smaller sample size and lower SNP density of the former. There is a moderate correlation of the Rsb distribution between the two datasets (Figure 2C).

### Genome Evidence of Departures from Neutrality

Do regions with extreme ln(Rsb) values indicate local selection, or the extremes of the neutral distribution? To answer this question without accurate information concerning the demographic history of the populations, the ideal statistic would be robust against violations of the neutral assumption due to demographic history, yet at the same time sensitive to departures from neutrality due to selection. Previous studies have shown via simulations that neutral distributions are heavily influenced by demographic parameters; exploration of a broad, neutral parameter space is necessary, but results are often ambiguous [21,24]. Nonetheless, an important feature shared by all demographic factors (but not selection) is that varying demographic parameters are expected to influence all regions of the genome equally; we therefore hypothesized that the shape of any neutral distribution should have certain invariant properties. More specifically, the density distributions of the standard transformed ln(Rsb) should show little change across a broad range of neutral parameters and varying ascertainment settings.

To investigate this, we generated a series of neutral demographic models using coalescent simulation. These include the simplest constant population-size model (model 1) and models with: different recombination rates (model 2) or mutation rates (model 3); independent ancestors for Europeans and Chinese (model 4); two different bottleneck scenarios (models 5 and 6, see Methods); population expansion (model 7); and population structure (model 8). We also considered three complex models matching real data in multiple aspects—one with no migration (model 9), one with migration and uniform recombination rate (model 10), and one with no migration but heterogeneous recombination rates (model 11) [35]. Ascertainment analogous to that of the Perlegen data, but much simplified, was applied to these models. (see Methods). Three other ascertainment processes were also simulated for model 10 (hereafter referred to as the standard neutral model because it is used as the null distribution to compare to the empirical data) to assess the impacts of low-quality sequencing (model 10-asc1), skewed constitution in the diversity panel (model 10-asc2), and high genotyping failure rate (model 10-asc3, see Methods for details on all models). It should be noted that the ascertainment schemes we use here do not capture the full complexity of the empirical data. Nonetheless, they provide perspectives as to how ascertainment in general affects ln(Rsb) distribution.

**Figure 3:**
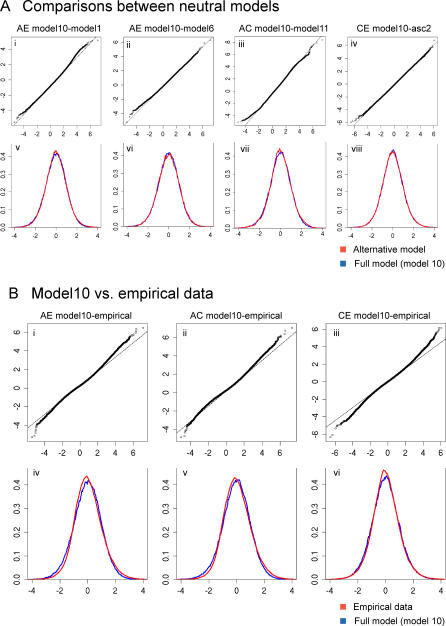
Comparisons of the ln(Rsb) Distributions between Various Neutral Simulations and between the Full Neutral Model and Empirical Data (A) shows the full neutral model (model 10) and several other neutral models; (B) shows model 10 and empirical data. The ln(Rsb) distributions are compared by both a QQ plot and superimposing standardized histograms. In every QQ plot, quantile points of model 10 are plotted on the *x*-axis. For all of the superimposed standardized histogram plots, the blue color designates the full model (model 10) and the red color designates the alternative model.

**Figure 4:**
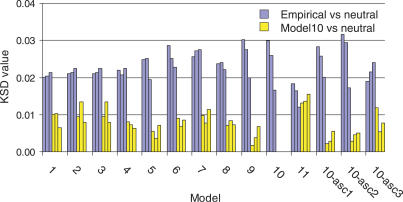
KSD Statistic for the Comparisons of Standardized ln(Rsb) Distributions within Neutral Models, and between Neutral Models and Empirical Data Categories on the *x*-axis denote the neutral models 1 to 10-asc3, where 10-asc1, 10-asc2, and 10-asc3 indicate the three different ascertainment schemes (see Methods). Each neutral distribution is compared to the corresponding empirical (in blue) or model 10 (in yellow) distribution. A cluster of three bars in the same color denotes the KSD values in the three pairwise comparisons in the order AE, AC, and CE.

Data from the neutral simulations described above, as well as the Perlegen data, were compared to the standard neutral model (model 10) by three approaches, namely quantile–quantile plots (QQ plots), superimposing histograms, and the Kolmogorov-Smirnov D (KSD) statistic (see Methods). The QQ plot shows a straight line when two distributions can be linearly transformed into each other. Nonstandard ln(Rsb)′ distributions were compared in pairwise fashion using QQ plots. For superimposing the histogram, the distributions of standard transformed ln(Rsb) were superimposed onto one another using the same coordinates (Figures 3 and S1–S13). The KSD value was also determined for the pairwise comparisons of the ln(Rsb) distributions as a quantitative measure of difference between distributions (Figure 4). The *p*-values for the Kolmogorov-Smirnov test are omitted due to the lack of independence between SNPs**.**

The QQ plots indicate that, as hypothesized, varying the neutral parameters does not heavily influence the shape of the density distributions (Figures 3 and S1–S13). Most models showed good agreement with the standard model (always plotted on the *x*-axis), except in some cases, such as the comparisons to the models with simple assumptions (models 1, 2, and 3), in which departures from linearity are more obvious at both ends of the QQ plot curve (Figures S1–S13). This is to be expected because the demographic assumptions of models 1, 2, and 3 differ very much from the standard model, and also because QQ plots of bell-shaped distributions have fewer data points and, therefore, more fluctuation at the tails of the curve. On the other hand, comparisons between empirical data and the standard neutral model showed that the QQ plots clearly departed from linearity even in the central segment, indicating a difference between the empirical data and the standard model that seemingly is not accounted for by demographic factors.

Histograms superimposing and KSD values show similar trends. Most ln(Rsb) distributions for the different neutral models with varying demographic parameters match very well with the standard model, except a few with noticeable mismatches, e.g., the models with different recombination and mutation rates (models 2 and 3) in the AC comparison (KSD = 0.0134 in both cases), and the model with heterogeneous recombination rate (KSD = 0.0131, 0.0136, and 0.0155 for AE, AC, and CE comparisons, respectively). However, superimposing the distributions for the empirical data and the standard model revealed much more pronounced differences: the modes of the empirical EA and CA distributions shift to the left, although with larger right tails compared to the simulated data; the empirical CE distribution is more symmetrical, but also shows larger tails at both ends than the neutral distribution (Figure 3). The KSD values for each neutral model against the empirical data generally exceed the corresponding comparisons against the standard model by a factor of two, with the majority (34/42) ranging between 0.02 and 0.0316 (Figure 4).

One exception is with heterogeneous recombination rates (model 11), in which the KSD values against the empirical data exceed only slightly, and in the case of the CE comparison actually fall below, the KSD value for the simulated model versus the standard model. This shows that ln(Rsb) is robust towards most demographic factors, but relatively sensitive to heterogeneous recombination rate (i.e., recombination hotspots). Nonetheless, we found that the difference in ln(Rsb) distributions between the recombination hotspot model and the uniform-recombination model does not come from the tail part of the distribution, which determines the robustness of the false-positive rate. In fact, for the right tail of the distribution, when we fix the false-positive rate of the standard model to be 0.5%, and apply the corresponding critical value to model 11, 0.71%, 0.77%, and 0.66% of SNPs are significant for the AC, AE, and CE comparisons, respectively, which are not drastically elevated from 0.5%.

### Defining Cutoffs for Top SNPs

In general, to determine cutoff values corresponding to a particular significance level, an empirical distribution should be compared to a null distribution by matching the neutral part to the null distribution. Here we simply matched the first and third quartile points of the empirical and the standard neutral model distributions. The resulting superimposed distributions resemble those of the ln(Rsb) histogram superimposition (Figures 3B and S14): For the AC and AE comparisons, we infer that the excess in the right tail of either distribution indicates the signal of positive selection in the non-African populations, whereas an excess in the left tail would indicate positive selection in the Africans. The two empirical distributions, when compared to their neutral distribution, have their modes shifted towards the left with a bigger tail at the right end, suggesting an even larger departure from the null distribution for the non-Africans if the modes are matched. There is a corresponding deficit in the left tail, obviously underevaluating the signals in Africans. In the CE comparison, although both the left and right tails extend farther than the null distribution, the empirical distribution has lower shoulders than the null distribution. Overall, these observations suggest that our approach is conservative for identifying ln(Rsb) values that truly deviate from neutral expectations.

We defined the level of significance to be 0.005 for the AE and AC comparisons and 0.001 for the CE comparison so as to make the cutoffs for the empirical distribution approximately two to three times that of the level of significance. The cutoffs thus defined are 1.35% for Europeans in the EA comparison, 1.14% for Chinese in the CA comparison, 0.303% for Europeans in the CE comparison, and 0.245% for Chinese in the CE comparison. Although the African group is also expected to have experienced local positive selection, our method down-weights any signals of selection in Africans, resulting in cutoff values for Africans that are even lower than the test level. Evaluation of positive selection in Africans, therefore, is not possible by this approach. It should be noted that, given the conservative nature of our approach, the cutoffs thus defined may not precisely estimate the fractions of the human genome under the effect of recent positive selection. However, in contrast to any arbitrary cutoffs, this conservative approach should provide a lower boundary for the distribution of recent, local positive selection. A better strategy, one that precisely matches the neutral part of the empirical data to the null hypothesis, is needed in the future to investigate the signal of positive selection in Africans as well as to provide more accurate cutoffs for non-Africans.

### Determining Candidate Regions

To further reduce noise in the data, the variance of ln(Rsb) values was estimated by bootstrapping across the genotyping panels. SNPS with ln(Rsb) values above the cutoff values (top SNPs, or tSNPs) but with high bootstrap ln(Rsb) variances were removed from further analysis (see Methods). The remaining tSNPs tend to cluster tightly into discrete regions, marked as sharp peaks in the Rsb map (Figure 1), which is expected from positive selection because the flanking SNPs are then influenced by hitchhiking.

In order to define candidate regions, we connected tSNPs within 200 kilobases (kb) of each other into discrete regions. Regions that span over 40 kb (half of the genome has linkage disequilibrium [LD] blocks of size 40 kb or longer for Europeans and Asians [36]) and that include more than 14 tSNPs were considered candidate regions and were extended 50 kb in both directions (50 kb is roughly the distance LD decays by half). We defined candidate regions for the HapMap and Perlegen data separately, each consisting of four candidate region lists, namely Eur-AE, Chn-AC, Eur-CE, and Chn-CE, where Eur-AE, for example, indicates candidate regions exhibiting a signature of selection in Europeans when compared to Africans. The two candidate region sets defined in the HapMap and Perlegen datasets were also combined into a common candidate region set, by merging overlapping regions and including those that encompass any tSNPs in the alternative dataset. Figure 1 shows one candidate region in the HapMap data, which contains the *SLC24A5* gene, a pigmentation gene for which there is prior evidence of selection in Europeans [12].

Table 1 lists examples of candidate regions from the common candidate region set with strong signals of local selection and the genes these regions contain. Complete lists of candidate regions are given in Tables S2–S13. The numbers of candidate regions from the HapMap data (in the order of Eur-AE, Chn-AC, Eur-CE, and Chn-CE) are: 298, 240, 62, and 49, respectively, roughly double that of the Perlegen data (147, 115, 25, and 22 regions, respectively), and also more than that of the combined candidate region set (216, 143, 23, and 19 regions, respectively). The sizes of the candidate regions in the combined set are on average 391 kb, 405 kb, 305 kb, and 339 kb for the Eur-AE, Chn-AC, Eur-CE, and Chn-CE comparisons, respectively, and range from 140 kb to 1,893 kb. The fact that the AE and AC comparisons produced many more candidate regions than the CE comparison is consistent with the notion that a common ancestral population of Europeans and Chinese diverged much earlier from Africans before subsequently splitting.

**Table 1:**
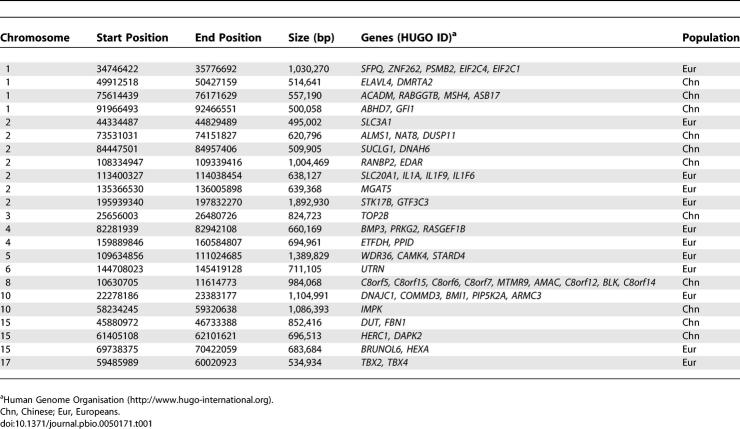
Some Examples of Candidate Regions with Strong Signals

### Power of Tests Based on ln(Rsb)

To evaluate the power of ln(Rsb)-based tests, we performed simulations of selective sweeps using the two-population model described in Thornton and Jensen [37], in which a derived population experiences a bottleneck while splitting from an ancestral population, and then selection occurs in the derived, but not the ancestral, population. Loci that were 2 megabases (Mb) long were generated for both neutral and selection scenarios (see Methods). To allow EHH to decay substantially before reaching the ends of loci, ln(Rsb) values were calculated only for SNPs residing in the central 1Mb.

The first test simply uses the ratio of tSNP over the total number of SNPs in each central 1-Mb region as the test statistic. The tSNP cutoff value is set to be the ln(Rsb) value that defines the top 0.5% tail area of the neutral distribution. SNPs with ln(Rsb) value greater than this value are assigned as tSNPs, and loci with a ratio of tSNPs above a critical value are called significant.

The second test is analogous to the ad hoc procedure used to define candidate regions in the empirical data. In this test, tSNPs are defined as above, and tSNPs within 200 kb of each other are connected into regions. A region is called a candidate region if it is longer than 40 kb in physical distance and contains more than 84 tSNPs (based on the 14 tSNP criterion used for the empirical Perlegen data, adjusted for the different SNP density in the simulated data). An accepted candidate region is extended 50 kb farther from both ends.

**Figure 5:**
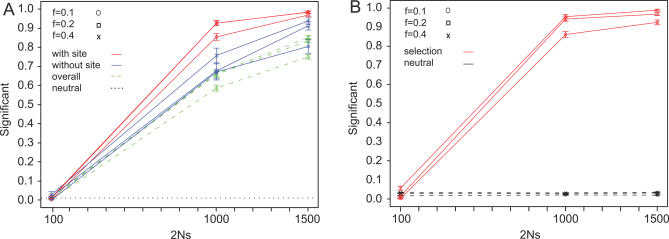
Power of the Two ln(Rsb)-Based Tests for Different Values of *f* (the Strength of the Bottleneck Experienced by the Derived Population) (A) The power plot for test I, which uses ratio of tSNPs as the test statistic (see Methods). The critical value is set by independent neutral simulation at a level of 0.01. Curves in red show the power to detect selection that occurs in the central 1 Mb. Curves in blue show the power to detect selection that occurs outside the central 1 Mb, but within 200 kb. Dashed curves in green are the overall power for the entire simulated 2-Mb segment. (B) The power plot for test II, the ad hoc test. A case is called significant only if the selected site lies within a candidate region.

Figure 5 is the power plot for both tests, at a false-positive rate of 0.01 for the first test. Both tests have almost no power to detect weak selection (alpha = 100). However, when the selection coefficient is relatively high (alpha = 1,000 and 1,500), they both show high power: for test 1, the power ranges from 0.85 to 0.98 when the selected site is in the central 1 Mb of the simulated locus; from 0.67 to 0.94 if the selected site lies within 200 kb outside of the central 1Mb region, and from 0.59 to 0.85 for the entire 2-Mb locus. For test 2, the power ranges from 0.86 to 0.96 for alpha = 1,000 and 1,500. Test 2 has a varying, but low, false-positive rate around 0.02 for *f* = 0.1, 0.029 for *f* = 0.2, and 0.033 for *f* =0.4, where *f* is a measure of the severity of the bottleneck experienced by the derived population (see Methods). The power of the test tends to be negatively correlated with the severity of bottleneck, which is to be expected since more severe bottlenecks create more fluctuations and also decrease the genetic diversity of the neutral loci. Notably, our tests have comparable power to the composite-likelihood test and its related tests, which are generally considered to be the best of the available tests [20,38,39]

### Overlap of Signals between the Perlegen and HapMap Datasets

There is significant overlap in the candidate regions from the HapMap and Perlegen datasets. Pairwise comparisons revealed 8%–40% overlap of the corresponding candidate region lists between the two sets (*p* < 0.001, Table 2). The concordance between the candidate regions identified in the HapMap and Perlegen datasets, although significant, is not very high, which is consistent with the moderate correlation of the ln(Rsb) distribution between the two datasets (Figure 1). This may in part reflect differences between these two datasets in SNP density, SNP discovery scheme, ascertainment, and sample size. Stochastic factors can also produce fluctuations in the magnitude of signals across different datasets, whereas conservative cutoffs decrease the chances of rediscovering the same regions. However, the biggest difference may reflect different population groups, especially in the case of Africa: the Perlegen samples consist of African Americans, who have admixed substantially with Europeans [40,41], thereby reducing the signature of selection when comparing African Americans with Europeans, whereas the HapMap sample consists of Yorubans from Nigeria, and hence are more representative of African genetic diversity. In fact, there is more overlap between the Perlegen and HapMap data for AC comparisons than for AE comparisons (fractions of overlap for the Eur-AE and Chn-AC lists are 0.13 and 0.19 in the HapMap data, and 0.26 and 0.40 in the Perlegen data, respectively, Table 2), which probably reflects the fact that the AC comparisons are less influenced by the European admixture in African Americans. Overall, the signals of selection from the HapMap data are probably more reliable because, aside from including native Africans, the HapMap data also has higher SNP density and larger sample sizes.

**Table 2:**
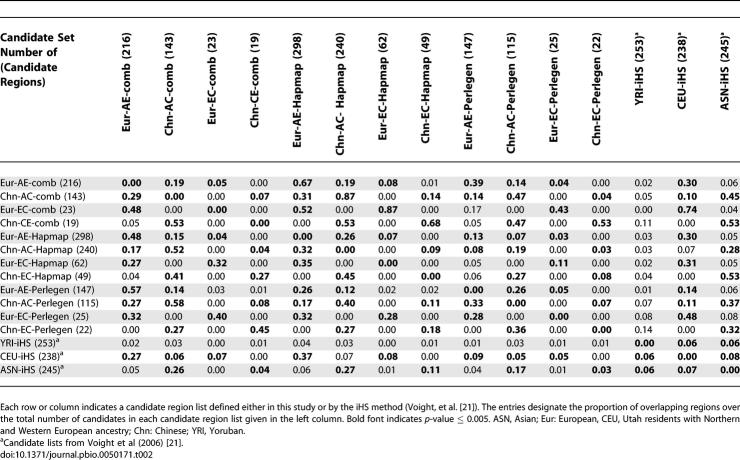
Pairwise Overlapping Test of Different Candidate Region Lists of Positive Selection

### Overlap within Candidate Region Sets

Table 2 shows that the overlap profile between candidate region lists, e.g., Eur-AE and Eur-CE, are similar across the HapMap, Perlegen, and combined sets: there is extensive (*p* < 0.005) overlapping between the Eur-AE and the Chn-AC regions, supporting the notion that a common population ancestral to Europeans and Asians experienced shared selection pressures before or during the out-of-Africa migration. On the other hand, substantial numbers of candidate regions from the CE comparisons overlap with regions from the same populations in the AE or AC comparisons, e.g., Eur-AE or Chn-AC (28% ~ 53%), but are almost absent from the lists of the opposite population (0% ~ 5%). Such signals are population specific and likely happened after the divergence of the two non-African populations.

### Overlapping of ln(Rsb) Signals with LRH and Integrated Haplotype Score Signals

Although our approach is designed to be most powerful for sweeps at or near fixation, it is interesting to see whether the method detects signals of partial sweeps as well. To investigate this, a long-range haplotype (LRH) test was constructed and applied to the ln(Rsb) candidates (see Methods). The LRH test assigns three *p*-values (*p*_75_, *p*_95_, and *p*_mean_) based on different criteria to each candidate region (see Methods). A high proportion of candidate regions are significant for positive selection under the LRH test, ranging from 0.193 to 0.556 in different candidate region sets (Table S1).

**Figure 6:**
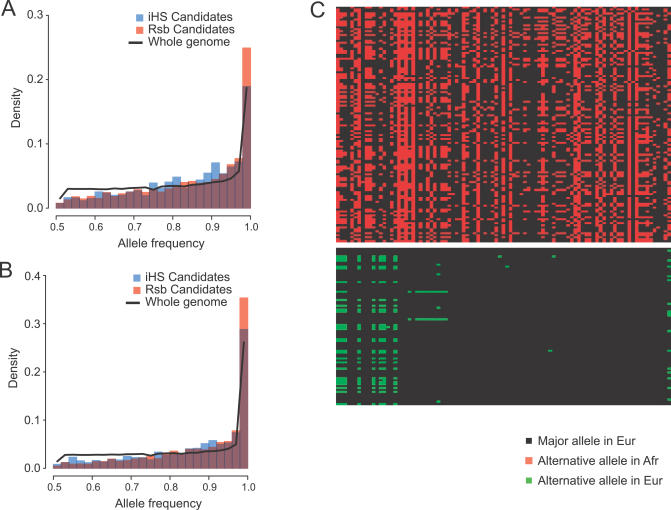
Comparisons of the Major Allele Spectra between the iHS and Rsb Candidate Regions, and the Genetic Diversity Pattern in the *SLC24A5* Region (A and B) The frequency spectra of the major alleles for SNPs are compared between the iHS candidate regions (in blue) and the 100-kb regions around the centers of the Rsb candidate regions (in orange) in either (A) Europeans or (B) Chinese. Overlapping parts are in dark purple. For comparison, the corresponding major allele–frequency spectra for the whole genome are shown as black curves. (C) shows, as an example, the genetic diversity pattern for the 200-kb region around the *SLC24A5* gene in Africans (Afr; upper section) and in Europeans (Eur; lower section), from the HapMap phase I data. Rows denote individual chromosomes, and columns denote the SNP sites.

We also determined the overlap between candidate regions defined by the ln(Rsb) statistic to those identified previously by the integrated haplotype score (iHS) statistic, which was shown to be most powerful for detecting partial sweeps [21]. A significant percentage of the candidate regions from the ln(Rsb) method overlap with candidate regions identified by the iHS method (30% ~ 74%, *p* < 0.001, Table 2). Given this substantial overlap, are there any differences in the properties of the ln(Rsb) and iHS candidate regions? To investigate this, we compared the major allele frequency spectra of SNPs between the iHS candidate regions, which are all 100 kb in size, and the 100-kb intervals around the centers of the ln(Rsb) candidate regions (Figure 6). The Perlegen genotype data were used for this comparison because of the more consistent ascertainment scheme. The allele frequency spectra of both iHS and Rsb regions differ substantially from that of the whole genome (Figure 6), exhibiting a deficiency of intermediate-frequency alleles and an excess of common/rare variants, a hallmark signature of positive selection. The Rsb spectra also deviate from the iHS spectra, with a slight excess of alleles near fixation (0.9 < allele frequency < 1) and a much higher abundance of fixed alleles (allele frequency = 1); on the other hand, iHS tends to have an excess of alleles between frequency 0.7 and 0.9, which is particularly evident in the European comparison (Figure 6). This clearly demonstrates the different specificity of these two methods: the ln(Rsb) method detects more fixed and nearly fixed selective sweeps, whereas the iHS approach has higher power to detect partial sweeps. It should be noted that the frequency of fixed alleles in the iHS spectra does not differ from that of the whole genome, whereas the frequency of fixed alleles in the ln(Rsb) spectra greatly exceeds that of the whole genome. This suggests that there may be a high proportion of recent sweeps that have reached fixation in individual populations.

### Genes in the Candidate Regions

One important question is whether signatures of selection are enriched in genic versus non-genic regions. We used the Refseq gene coordinates to define genic regions; because the genic regions thus defined constitute a large proportion (~1/3) of the whole genome, we asked whether our candidate regions span more genic regions than expected by chance (Methods). The fraction of the total physical distance encompassed by the candidate regions that are genic ranges between 0.317 and 0.420 for different candidate region lists. For most cases, there is neither an excess nor a deficit of genic regions in the candidate regions, except there is a significant enrichment of genic regions in the EUR-CE candidate regions from the Perlegen data (54.8%, *p* = 0.005). This seemingly disagrees with the expectation that positive selection should happen more often in genes. One possible explanation is that our method detects selection sweep patterns that influence a large interval around the causal locus, which may not be gene rich. Another possibility is that regulatory regions outside genes might also have experienced substantial recent positive selection.

Our approach captured some genes that have previously been shown to exhibit strong signatures of positive selection. These include drug-metabolizing genes *CYP3A4* and *CYP3A5* (Europeans [9]), skin pigmentation genes *MYO5A* (Europeans [21,42]) and *SLC24A5* (Europeans [12]), immune system transcription factor interleukin 4, sepsis resistant gene *Caspase12* (Chinese, Europeans [11]), the lactose tolerance gene (Europeans [8]), and the dietary calcium absorption-related ion channel gene *TRPV6* (Chinese, Europeans [43,44]). Rediscovery of such positive examples supports the high power of our approach. When looked at in more detail, these genes all show the pattern expected for sweeps near to fixation. It is also notable that the sweep signals of two genes, *CASP12* and *SLC24A5,* were not detected by the iHS approach [21], but are among the strongest signals found in our study.

An intriguing question is what kinds of genes were involved in recent, local positive selection and therefore have had a potentially strong impact on recent human evolution. We performed a gene ontology (GO) analysis to determine whether any categories of genes are overrepresented in our candidate regions (Table 3, Methods). One GO category, interleukin-1 receptor antagonist activity, is found to be highly significantly overrepresented (raw *p*-values = 4.62 × 10^−9^, 2.94 × 10^−12^. When controlled for multiple testing, the false discovery rate [FDR] is 0.0021). However, all six of the genes in this GO category cluster in a 400-kb region in Chromosome 2. It is therefore not clear whether the signal of local selection comes from one or from multiple genes in this GO category. Other categories that show significant enrichment include those for genes involved in growth factor activity, cytokine activity, monooxygenase activity, ceramidase activity, calmodulin binding, various transporter and cofactor transporter activities, and vitamin transport. Cytokine and growth factor activity genes are involved in cell proliferation, development, and bone morphology, which might contribute to morphological differences between groups. Significant GO groups, such as cofactor transporter activity and vitamin transport, may have been involved in the adaptation to new food sources, or agriculture.

**Table 3:**
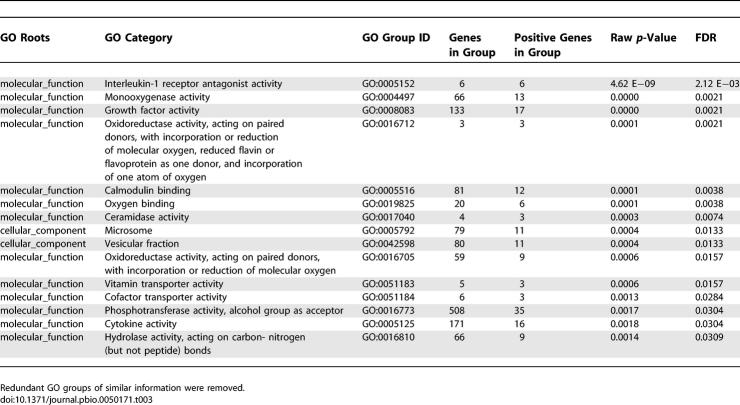
Summary of the Gene Ontology Analysis Results

In contrast to a previous study of local selection [21], we did not detect significant enrichment of gamatogenesis or related genes, nor did the olfaction or chemosensory perception genes emerge as overrepresented groups. This may reflect differences between the tests used in the GO analysis, but may also suggest that the selective pressures that acted on these functional groups differ, one type being more ancient or more dramatic and causing complete or nearly complete sweeps, the other representing more recent and/or weaker selection and hence partial sweeps. It should be noted, however, that GO analysis for genome candidate regions is biased and limited in power for several reasons. First, functionally related genes are often spatially close to each other; significant results may thus arise via physical association rather than independent selection. Second, in each GO category, there are usually a very small number of member genes, and the multiple testing corrections can easily mask already weak signals. Third, in each candidate region, there may be more than one gene, and it is not clear which one hosted the selection event (or, indeed, there may even be more than one selection event in a candidate region). To gain more insights, each gene should be checked individually.

## Discussion

Much progress has been made recently in developing methods to detect the signature of local positive selection in genomic data [18–23]. In this study, we offer three improvements and advances on existing methods. First, we have established a fast and simple counting algorithm to estimate EHH-derived statistics, such as iES, directly from dense genotype data from unrelated individuals without prior estimation of haplotype frequencies. The results of the counting algorithm are in good agreement with analytical estimation based on phased data. More importantly, with the counting algorithm, one can already achieve high test power for detecting recent, strong selective sweeps. Furthermore, by avoiding the intensive computation needed to estimate haplotypes from genotypic data, the counting algorithm provides superior flexibility for data updating, error checking, bootstrapping, and analyses of other sources of high-density SNP data, such as those produced by genotyping chips.

Second, distinguishing signals of selection from extreme patterns produced by neutrality has been a major obstacle in the study of positive selection; highly variable patterns given by certain demographic factors, such as bottlenecks and population structure, further obscure the signals [1,6]. Here, we show that by using the standard transformed ln(Rsb) statistic, neutral simulation distributions remained largely invariant over a wide range of demographic parameter values and various ascertainment schemes. The ln(Rsb) distribution thus is robust to departures from neutrality due to various demographic events or other factors that are expected to have an equal impact across the genome. Moreover, the observed standardized ln(Rsb) distributions departed markedly from neutral simulations, providing strong evidence that recent positive selection has indeed been occurring in our species. It should be emphasized that the method we used to match the empirical distribution to the neutral distribution (namely, matching the two quartile points) is conservative and masked any signals of selection in Africans. Nonetheless, we defined the signal cutoffs through a rigid statistical procedure, and they are thus likely to represent the lower boundary of the fraction of recent positive selection in the human genome.

Third, we introduce a new statistic, ln(Rsb), designed to complement existing statistics such as iHS [21]. The iHS statistic compares EHH between alternative alleles at a SNP, and hence has low power when one allele is at high frequency (and no power if an allele is fixed). However, the Rsb statistic, which compares EHH for the same SNP in two different populations, can provide evidence of selection in such cases. A power test demonstrates that tests based on ln(Rsb) possess high power for detecting strong selective sweeps that have reached fixation. Furthermore, our method detected numerous genes that have been previously shown to have strong evidence of selection, including *CYP3A, MYO5A, SLC24A5, IL4, CASP12*, and *LCT* [8,9,11,12,21,42,45], most of which are near or at fixation. It is especially notable that *SLC24A5* and *CASP12,* which were not detected by the iHS test, gave strong signals in the ln(Rsb) test, supporting the reliability and power of this approach. Nonetheless, our approach failed to detect some other genes that previously showed strong evidence of selection, such as *PDYN, MATP,* and the 17q21 inversion [13,46,47], probably because these genes/regions have been subject to partial sweeps. Nonetheless, both the LRH test and the overlap with iHS candidates revealed that our approach should have considerable power to detect even partial selection sweeps. One potential problem is that the ln(Rsb) statistic has low power when selection was shared by all human populations, e.g., before or during the initial migration out of Africa. A more comprehensive view on recent positive selection in our species should be obtained by combining signals and investigating candidates from this study with those from other approaches such as iHS [21].

Our approach has identified approximately 500 candidate regions in the combined list, after excluding redundancy from overlaps. However, it is very unlikely that there was a separate selection event for each of these signals; multiple genes controlling a complex trait, such as pigmentation, may have been co-selected. Strong single selection events might also influence multiple genes, thereby producing multiple candidate regions. There is also substantial overlapping of candidate regions between Chinese and Europeans (0.19 ~ 0.33), which further indicates either shared selection events or parallel selection. Analyzing interactions between genes under selection pressures should provide further insights into human evolution.

Finally, we detected some different GO groups that are enriched in our candidate regions. Some might help explain interpopulation differences in morphology, while others might have been involved in adaptation to novel food resources, pathogens, etc. Further detailed investigation of interesting candidate genes identified by this study should provide insights into the role and nature of adaptive events in the recent history of our species.

## Materials and Methods

### SNP databases.

We used two publicly available SNP datasets, the approximately 1.5 million SNPs from Perlegen Science [14] and the approximately 3.8 million SNPs from the international HapMap Project [15]. Perlegen identified SNPs mainly by resequencing a discovery panel of 24 individuals, and genotyped 23, 24, and 24 unrelated individuals from African Americans, European Americans, and Han Chinese, respectively. Ascertainment of HapMap SNPs was heterogeneous and complicated [48]; genotypes were obtained for family trios in both Yorubans and Europeans, and unrelated individuals from both Han Chinese and Japanese. For the evaluation of the counting algorithm and the LRH test, we used the HapMap phase I data, for which the whole-chromosome phases were provided for about 1 million SNPs. Only SNPs that overlapped among all four populations were included in the analyses.

### LRH test.

We used the HapMap phase I data, for which the haplotypes are available, for long-range haplotype (LRH) tests. The LRH statistic calculated here is similar to the iHS statistic introduced by [21]. Briefly, EHHA was first calculated for both alleles of a SNP, extending in both directions from the SNP until EHHA decreased to 0.05. The area under the EHHA curve was determined by integration to give the integrated EHHA (iEA), and the relative iEA within population (Raw) was calculated as:

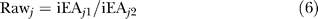
where the subscripts 1 and 2 denote the alternative alleles for SNP *j*. Singletons were removed from the analysis. Because the iEA calculation is sensitive to large gaps between successive SNPs, SNPs whose EHH decay plot hit gaps greater than 50 kb were removed from further analysis. The Raw values were then binned by major allele frequency. Within each bin, Raw values were ranked and assigned corresponding percentile values (LRHp). We then evaluated the percentages of SNPs with LRHp above the 5% or 25% threshold, and also the average LRHp for each candidate region, and derived three corresponding *p*-values: *p*_95_, *p*_75_, and *p*_mean_ by comparing them to 1,000 iterations of random genome regions of the same size.

### The iES calculation.

The calculation of the statistic iES starts by estimating EHHS around a core SNP as described in the Results. EHHS thus defined starts from one and decays towards zero as flanking sites are included that are progressively farther from the core SNP (Figure 2A). In this study, we calculated EHHS until it reached 0.1.

The iES statistic then integrates the area under the EHHS curve:

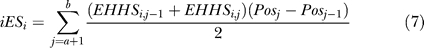
where *a* and *b* are the two ending positions where EHHS drops below 0.1, and *Pos_i_* is the physical position of site *j*.

### Treatment of missing data for the counting algorithm.

Missing genotypes were assigned as probability vectors conditioned on nearby genotypes as follows:

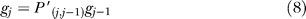
where *g_j_* is the missing genotype, *g_j−_*_1_ is the vector of genotype probabilities (*g*_11_, *g*_12_, *g*_22_) in the preceding site, e.g., taking (1, 0, 0) if *g_j_*_−1_ is homozygous A_1_A_1_ for the first allele, and *P*′_(*j,j*−1)_ is the matrix of conditional probability of genotype *j* given *j* − 1, defined by the non-missing data.

### Influence of recombination rate and SNP density.

Because iES is sensitive to large gaps in the distribution of SNPs, SNPs close to such regions were excluded from analysis. To identify such SNPs, gaps larger than 200 kb were identified, and if any iES value within 200 kb of a gap exceeded 300,000, then the gap boundary was extended by 200 kb and the procedure repeated until no extreme iES values were found. Gaps derived from the three different populations were merged to give universal gaps, and SNPs within these gaps were discarded.

To investigate how Rsb is influenced by varying recombination rates and SNP densities, Rsb was plotted against local recombination rate and SNP density, and correlations were calculated. Recombination rates were taken from the 1-Mb deCODE map [49]. SNP density was defined as the number of SNPs within a 20-kb window centered at each SNP.

### Neutral and positive selection simulations.

A series of neutral demographic models were simulated, ranging from the simplest constant population-size model to complex models matching real data in multiple aspects [35]. The basic model (model 1) used *N*_e_ =10,000 for all three populations, with the mutation rate μ and recombination rate γ assigned values of 1.5 × 10^−8^ and 1.3 × 10^−8^ site^−1^generation^−1^, respectively. The migration out of Africa and subsequent split between Asians and Europeans were set to be 3,500 and 2,000 generations ago, respectively. Based on this, further models doubled the recombination rate (model 2) or mutation rate (model 3), or split the ancestral population directly into three individual populations 3,500 generation ago (model 4). Two additional models added brief, severe bottlenecks immediately after population splitting (lasting for 100 generations, during which the population size shrank to 1%) either during the out-of-Africa migration and the origin of Europeans (model 5), or during the out-of-Africa migration and the origin of all three populations (model 6). The effect of population expansion was also studied by retaining the present population sizes and other configurations from the basic model while decreasing the population sizes back in time (model 7, present population sizes all set to 100,000; African population size changes to 24,000 at 200 generation ago; European and Chinese population sizes both change to 7,700 at 350 and 400 generations ago, respectively; and the ancient, common ancestor population size set to 12,500 at 17,000 generations ago). The effect of population structure was analyzed by modeling a hypothetical European population that split into two isolated groups (ratio of group sizes of 7:3) between 20 and 1,500 generations ago (model 8). Finally, we analyzed models featuring the complete best-fitting conditions, but one without migration and with uniform recombination rates (model 9), one with migration and uniform recombination rates (model 10, the standard model), and one without migration, but featuring heterogeneous recombination rates and recombination hotspots (model 11 [35]).

All of the above neutral models except model 11 were simulated with the ms coalescent simulation package [50]. Model 11, which uses heterogeneous recombination rates, was simulated with Cosi [35]. Two hundred fragments of 2 Mb were generated for each model, and only SNPs in the central 1 Mb were used for iES/ln(Rsb) calculations. The iES and ln(Rsb) calculations were carried out by first randomly pairing the simulated chromosomes to form pseudo-genotype data, and than applying the counting algorithm .

The ascertainment scheme approximating that of the Perlegen dataset was applied to the simulated data from models 1 through 10. Basically, the genotyping sample panels (23 Africans, 24 Chinese, and 24 Europeans) and discovery panel (six Africans, seven Chinese, and seven Europeans) were generated simultaneously, and each SNP from the genotyping sample was included only if it was polymorphic in the diversity panel. In addition, three different ascertainment processes were simulated for the standard mode. The first (model 10-asc1) assesses the influence of low-quality sequence data by using a very small discovery panel (two Africans, three Chinese, and three Europeans); the second (model 10-asc2) assesses a skewed constitution in the discovery panel (two Africans, four Europeans, and seven Chinese); the third model randomly eliminated 90% of the genotyping SNPs to match the empirical SNP density.

Simulations of selective sweeps were performed using the two-population model described in Thornton and Jensen [37]. This model simulates a selective sweep in a derived population that has experienced a population bottleneck, and also generates data from an ancestral, equilibrium population. The bottleneck parameters are *t*_r_, the time at which the population recovered from the bottleneck, *d,* the duration of the bottleneck, and *f,* the relative size of the population during the bottleneck. The parameters *t*_r_ and *d* are measured in units of 4N_e_ generations. The sweep parameters are, α = 2N_e_s, the strength of selection, *X,* the location of the selected site, and τ, the time in the past when the beneficial allele reached fixation in the population. Genome scan datasets consisting of 100 loci were simulated, with ten loci experiencing a selective sweep. Loci were 2 Mb in length, with θ = 0.001/site, and ρ = 4N_e_r = 0.00052/site. For loci experiencing a sweep, *X* was assigned uniformly at random along the 2 Mb. The parameters *t*_r_, *d,* and τ were fixed at 0.0075, 0.055, and 0.0076, respectively. We varied *f* to represent bottlenecks of different severity, with *f* = 0.1, 0.2, and 0.4. The strength of selection was varied from alpha = 100, 1,000, and 1,500. The cutoff for tSNPs and the critical value of the ratio of tSNPs in test 1 were obtained by simulating 5,000 independent neutral loci as above for each *f* = 0.1, 0.2, and 0.4. For both the ancestral and the derived populations, we sampled 60 chromosomes. The ancestral population was used to represent Africans and the derived to represent an out-of-Africa population. Ascertainment is similar to the neutral simulation, except there are only two populations. The discovery panel used 12 chromosomes from either population, and either genotyping panel included 48 chromosomes.

### Defining cutoffs.

The full model (model 10) distribution and the empirical data were transformed and superimposed by matching the first and third quartile points as follows:

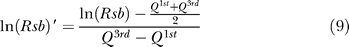
where *Q* ^1st^ and *Q* ^3rd^ are the first and third quantile values of ln(Rsb). The neutral model was treated as the null hypothesis, and threshold values were positioned on the null distribution at the desired significance levels.

### Noise reduction by bootstrapping.

The full Perlegen and HapMap genotyping panels were bootstrapped over individuals for 50 times each, and iES and Rsb were calculated for each SNP in each bootstrap resample, providing an estimated standard deviation of the ln(Rsb) value for every SNP. As inconsistent signals are more likely to come from SNPs with large variances in ln(Rsb), we constructed the distribution of SD(ln(Rsb))/ln(Rsb) for SNPs above the cutoffs, and discarded those SNPs in the bottom 50% of this distribution. The remaining SNPs are defined as tSNPs**.**

### Testing enrichment of evidence for selection in genic regions.

We investigated whether genic regions exhibit more evidence of local selection than non-genic regions. We calculated the proportion of the full length of candidate regions in each candidate region list that overlapped genes, and obtained *p*-values by comparing the observed proportions to those observed in 1,000 samples of random genome regions of the same size.

### Overlapping of candidate regions between datasets.

Pairwise overlapping was calculated between the candidate regions from the HapMap, Perlegen, and combined datasets. We also checked the overlapping of our combined candidate regions against a previous genome scan for positive selection [21]. Significance of the number of overlapping regions was evaluated by comparing the observed overlap to 1,000 iterations of random samples of two sets of regions with the same size as the candidate regions.

### Gene ontology analysis.

Candidate regions obtained from the HapMap and Perlegen data were merged to produce a single universal set of genomic regions exhibiting evidence of recent local selection. This list was used to investigate the types of genes involved in local selection. We used the FUNC GO analysis package for this purpose (http://func.eva.mpg.de [51]). BioMart (http://www.biomart.org/index.html) was used to assign GO IDs for genes listed in the ENSEMBL known-gene database. The AmiGO gene ontology database (http://amigo.geneontology.org) was used to construct the GO tree. The hypergeometric test was used to find GO categories that are overrepresented in the candidate regions.

## Supporting Information

Figure S1 Comparisons of the log(Rsb) Distributions between Neutral Model 1 and Neutral Model 10 Upper row: the QQ plots. In every QQ plot, quantile points of model 10 are plotted on the *x*-axis. Lower row: standardized ln(Rsb) histograms are superimposed onto one another. The blue curve designates model 10, and the red curve designates model 1.
(99 KB PPT)

Figure S2 Comparisons of the log(Rsb) Distributions between Neutral Model 2 and Neutral Model 10 Upper row: the QQ plots. In every QQ plot, quantile points of model 10 are plotted on the *x*-axis. Lower row: standardized ln(Rsb) histograms are superimposed onto one another. The blue curve designates model 10, and the red curve designates model 2.
(71 KB PPT)

Figure S3 Comparisons of the log(Rsb) Distributions between Neutral Model 3 and Neutral Model 10 Upper row: the QQ plots. In every QQ plot, quantile points of model 10 are plotted on the *x*-axis. Lower row: standardized ln(Rsb) histograms are superimposed onto one another. The blue curve designates model 10, and the red curve designates model 3.
(71 KB PPT)

Figure S4 Comparisons of the log(Rsb) Distributions between Neutral Model 4 and Neutral Model 10 Upper row: the QQ plots. In every QQ plot, quantile points of model 10 are plotted on the *x*-axis. Lower row: standardized ln(Rsb) histograms are superimposed onto one another. The blue curve designates model 10, and the red curve designates model 4.
(70 KB PPT)

Figure S5 Comparisons of the log(Rsb) Distributions between Neutral Model 1 and Neutral Model 10 Upper row: the QQ plots. In every QQ plot, quantile points of model 10 are plotted on the *x*-axis. Lower row: standardized ln(Rsb) histograms are superimposed onto one another. The blue curve designates model 10, and the red curve designates model 5.
(70 KB PPT)

Figure S6 Comparisons of the log(Rsb) Distributions between Neutral Model 6 and Neutral Model 10 Upper row: the QQ plots. In every QQ plot, quantile points of model 10 are plotted on the *x*-axis. Lower row: standardized ln(Rsb) histograms are superimposed onto one another. The blue curve designates model 10, and the red curve designates model 6.
(70 KB PPT)

Figure S7 Comparisons of the log(Rsb) Distributions between Neutral Model 7 and Neutral Model 10 Upper row: the QQ plots. In every QQ plot, quantile points of model 10 are plotted on the *x*-axis. Lower row: standardized ln(Rsb) histograms are superimposed onto one another. The blue curve designates model 10, and the red curve designates model 7.
(70 KB PPT)

Figure S8 Comparisons of the log(Rsb) Distributions between Neutral Model 8 and Neutral Model 10 Upper row: the QQ plots. In every QQ plot, quantile points of model 10 are plotted on the *x*-axis. Lower row: standardized ln(Rsb) histograms are superimposed onto one another. The blue curve designates model 10, and the red curve designates model 8.
(70 KB PPT)

Figure S9 Comparisons of the log(Rsb) Distributions between Neutral Model 9 and Neutral Model 10 Upper row: the QQ plots. In every QQ plot, quantile points of model 10 are plotted on the *x*-axis. Lower row: standardized ln(Rsb) histograms are superimposed onto one another. The blue curve designates model 10, and the red curve designates model 9.
(70 KB PPT)

Figure S10 Comparisons of the log(Rsb) Distributions between Neutral Model 11 and Neutral Model 10 Upper row: the QQ plots. In every QQ plot, quantile points of model 10 are plotted on the *x*-axis. Lower row: standardized ln(Rsb) histograms are superimposed onto one another. The blue curve designates model 10, and the red curve designates model 11.
(73 KB PPT)

Figure S11 Comparisons of the log(Rsb) Distributions between Standard Ascertainment and the Alternative Ascertainment 1 for Model 10 Upper row: the QQ plots. In every QQ plot, quantile points of the standard ascertainment are plotted on the *x*-axis. Lower row: standardized log(Rsb) histograms are superimposed onto each other. The blue color designates the standard ascertainment for model 10, and the blue color designates the alternative ascertainment 1.
(70 KB PPT)

Figure S12 Comparisons of the log(Rsb) Distributions between Standard Ascertainment and the Alternative Ascertainment 2 for Model 10 Upper row: the QQ plots. In every QQ plot, quantile points of the standard ascertainment are plotted on the *x*-axis. Lower row: standardized log(Rsb) histograms are superimposed onto each other. The blue color designates the standard ascertainment for model 10, and the blue color designates the alternative ascertainment 2.
(71 KB PPT)

Figure S13 Comparisons of the log(Rsb) Distributions between Standard Ascertainment and the Alternative Ascertainment 3 for Model 10 Upper row: the QQ plots. In every QQ plot, quantile points of the standard ascertainment are plotted on the *x*-axis. Lower row: standardized log(Rsb) histograms are superimposed onto each other. The blue color designates the standard ascertainment for model 10, and the blue color designates the alternative ascertainment 3.
(70 KB PPT)

Figure S14 The Empirical ln(Rsb) Distributions Superimposed onto the Null Distribution to Define the Cutoffs The empirical ln(Rsb) distributions of the AE, AC, and CE comparisons (a, b, and c) were matched to their corresponding null distributions, defined by model 10, by the first and third quartile points. The blue curves designate the null distribution, and the red curves designate the empirical data. The cutoff positions are indicated by the short vertical lines.
(59 KB PPT)

Table S1 Proportions of Candidate Region Lists That Are Significant in the LRH Test Pmn, P95, and P75 are related LRH tests that use the mean, number of SNPs above 95%, or number of SNPs above 75% as the test statistics, respectively (see Methods).
(36 KB DOC)

Table S2 Candidate Regions for Europeans from the AE Comparison in the HapMap Data (37 KB XLS)

Table S3 Candidate Regions for Chinese from the AC Comparison in the HapMap Data (32 KB XLS)

Table S4 Candidate Regions for Europeans from the CE Comparison in the HapMap Data (24 KB XLS)

Table S5 Candidate Regions for Chinese from the CE Comparison in the HapMap Data (22 KB XLS)

Table S6 Candidate Regions for Europeans from the AE Comparison in the Perlegen Data (37 KB XLS)

Table S7 Candidate Regions for Chinese from the AC Comparison in the Perlegen Data (34 KB XLS)

Table S8 Candidate Regions for Europeans from the CE Comparison in the Perlegen Data (19 KB XLS)

Table S9 Candidate Regions for Chinese from the CE Comparison in the Perlegen Data (18 KB XLS)

Table S10 Combined Candidate Regions for Europeans from the AE Comparisons in the Perlegen and HapMap Data (41 KB XLS)

Table S11 Combined Candidate Regions for Chinese from the AC Comparisons in the Perlegen and HapMap Data (41 KB XLS)

Table S12 Combined Candidate Regions for Europeans from the CE Comparisons in the Perlegen and HapMap Data (20 KB XLS)

Table S13 Combined Candidate Regions for Chinese from the CE Comparisons in the Perlegen and HapMap Data (19 KB XLS)

## Abbreviations

AC: African versus Chinese population
AE: African versus European population
CE: Chinese versus European population
EHH: extended haplotype homozygosity
EHHA: extended haplotype homozygosity of an allele
EHHS: extended haplotype homozygosity of a single nucleotide polymorphism site
GO: gene ontology
iES: integrated extended haplotype homozygosity of a single nucleotide polymorphism site
iHS: integrated haplotype score
kb: kilobases
KSD: Kolmogorov-Smirnov D statistic
LRH: long-range haplotype
Mb: megabase
QQ plot: quantile–quantile plot
SNP: single nucleotide polymorphism
tSNP: top single nucleotide polymorphism

## References

[pbio-0050171-b001] Bamshad M, Wooding SP (2003). Signatures of natural selection in the human genome. Nat Rev Genet.

[pbio-0050171-b002] Bowcock AM, Kidd JR, Mountain JL, Hebert JM, Carotenuto L (1991). Drift, admixture, and selection in human evolution: A study with DNA polymorphisms. Proc Natl Acad Sci U S A.

[pbio-0050171-b003] Kreitman M (2000). Methods to detect selection in populations with applications to the human. Annu Rev Genomics Hum Genet.

[pbio-0050171-b004] Nielsen R (2005). Molecular signatures of natural selection. Annu Rev Genet.

[pbio-0050171-b005] Ronald J, Akey JM (2005). Genome-wide scans for loci under selection in humans. Hum Genomics.

[pbio-0050171-b006] Sabeti PC, Schaffner SF, Fry B, Lohmueller J, Varilly P (2006). Positive natural selection in the human lineage. Science.

[pbio-0050171-b007] Vallender EJ, Lahn BT (2004). Positive selection on the human genome. Hum Mol Genet.

[pbio-0050171-b008] Bersaglieri T, Sabeti PC, Patterson N, Vanderploeg T, Schaffner SF (2004). Genetic signatures of strong recent positive selection at the lactase gene. Am J Hum Genet.

[pbio-0050171-b009] Thompson EE, Kuttab-Boulos H, Witonsky D, Yang L, Roe BA (2004). CYP3A variation and the evolution of salt-sensitivity variants. Am J Hum Genet.

[pbio-0050171-b010] Saunders MA, Hammer MF, Nachman MW (2002). Nucleotide variability at G6pd and the signature of malarial selection in humans. Genetics.

[pbio-0050171-b011] Xue Y, Daly A, Yngvadottir B, Liu M, Coop G (2006). Spread of an inactive form of caspase-12 in humans is due to recent positive selection. Am J Hum Genet.

[pbio-0050171-b012] Lamason RL, Mohideen MA, Mest JR, Wong AC, Norton HL (2005). SLC24A5, a putative cation exchanger, affects pigmentation in zebrafish and humans. Science.

[pbio-0050171-b013] Soejima M, Tachida H, Ishida T, Sano A, Koda Y (2006). Evidence for recent positive selection at the human AIM1 locus in a European population. Mol Biol Evol.

[pbio-0050171-b014] Hinds DA, Stuve LL, Nilsen GB, Halperin E, Eskin E (2005). Whole-genome patterns of common DNA variation in three human populations. Science.

[pbio-0050171-b015] The-International-HapMap-Consortium (2005). A haplotype map of the human genome. Nature.

[pbio-0050171-b016] Bonnen PE, Pe'er I, Plenge RM, Salit J, Lowe JK (2006). Evaluating potential for whole-genome studies in Kosrae, an isolated population in Micronesia. Nat Genet.

[pbio-0050171-b017] Nicolae DL, Wen X, Voight BF, Cox NJ (2006). Coverage and characteristics of the Affymetrix GeneChip Human Mapping 100K SNP set. PLoS Genet.

[pbio-0050171-b018] Carlson CS, Thomas DJ, Eberle MA, Swanson JE, Livingston RJ (2005). Genomic regions exhibiting positive selection identified from dense genotype data. Genome Res.

[pbio-0050171-b019] Kelley JL, Madeoy J, Calhoun JC, Swanson W, Akey JM (2006). Genomic signatures of positive selection in humans and the limits of outlier approaches. Genome Res.

[pbio-0050171-b020] Nielsen R, Williamson S, Kim Y, Hubisz MJ, Clark AG (2005). Genomic scans for selective sweeps using SNP data. Genome Res.

[pbio-0050171-b021] Voight BF, Kudaravalli S, Wen X, Pritchard JK (2006). A map of recent positive selection in the human genome. PLoS Biol.

[pbio-0050171-b022] Wang ET, Kodama G, Baldi P, Moyzis RK (2006). Global landscape of recent inferred Darwinian selection for Homo sapiens. Proc Natl Acad Sci U S A.

[pbio-0050171-b023] Zhang C, Bailey DK, Awad T, Liu G, Xing G (2006). A whole genome long-range haplotype (WGLRH) test for detecting imprints of positive selection in human populations. Bioinformatics.

[pbio-0050171-b024] Teshima KM, Coop G, Przeworski M (2006). How reliable are empirical genomic scans for selective sweeps?. Genome Res.

[pbio-0050171-b025] Sabeti PC, Reich DE, Higgins JM, Levine HZ, Richter DJ (2002). Detecting recent positive selection in the human genome from haplotype structure. Nature.

[pbio-0050171-b026] Sabeti PC, Walsh E, Schaffner SF, Varilly P, Fry B (2005). The case for selection at CCR5-Delta32. PLoS Biol.

[pbio-0050171-b027] Tang K, Wong LP, Lee EJ, Chong SS, Lee CG (2004). Genomic evidence for recent positive selection at the human MDR1 gene locus. Hum Mol Genet.

[pbio-0050171-b028] Walsh EC, Sabeti P, Hutcheson HB, Fry B, Schaffner SF (2006). Searching for signals of evolutionary selection in 168 genes related to immune function. Hum Genet.

[pbio-0050171-b029] Kauer MO, Dieringer D, Schlotterer C (2003). A microsatellite variability screen for positive selection associated with the “out of Africa” habitat expansion of Drosophila melanogaster. Genetics.

[pbio-0050171-b030] Schlotterer C (2002). A microsatellite-based multilocus screen for the identification of local selective sweeps. Genetics.

[pbio-0050171-b031] Excoffier L, Slatkin M (1995). Maximum-likelihood estimation of molecular haplotype frequencies in a diploid population. Mol Biol Evol.

[pbio-0050171-b032] Stephens M, Donnelly P (2003). A comparison of Bayesian methods for haplotype reconstruction from population genotype data. Am J Hum Genet.

[pbio-0050171-b033] Stephens M, Smith NJ, Donnelly P (2001). A new statistical method for haplotype reconstruction from population data. Am J Hum Genet.

[pbio-0050171-b034] Serre D, Nadon R, Hudson TJ (2005). Large-scale recombination rate patterns are conserved among human populations. Genome Res.

[pbio-0050171-b035] Schaffner SF, Foo C, Gabriel S, Reich D, Daly MJ (2005). Calibrating a coalescent simulation of human genome sequence variation. Genome Res.

[pbio-0050171-b036] Gabriel SB, Schaffner SF, Nguyen H, Moore JM, Roy J (2002). The structure of haplotype blocks in the human genome. Science.

[pbio-0050171-b037] Thornton KR, Jensen JD (2007). Controlling the false-positive rate in multilocus genome scans for selection. Genetics.

[pbio-0050171-b038] Kim Y, Stephan W (2002). Detecting a local signature of genetic hitchhiking along a recombining chromosome. Genetics.

[pbio-0050171-b039] Li H, Stephan W (2005). Maximum-likelihood methods for detecting recent positive selection and localizing the selected site in the genome. Genetics.

[pbio-0050171-b040] Patterson N, Hattangadi N, Lane B, Lohmueller KE, Hafler DA (2004). Methods for high-density admixture mapping of disease genes. Am J Hum Genet.

[pbio-0050171-b041] Smith MW, Patterson N, Lautenberger JA, Truelove AL, McDonald GJ (2004). A high-density admixture map for disease gene discovery in African Americans. Am J Hum Genet.

[pbio-0050171-b042] McEvoy B, Beleza S, Shriver MD (2006). The genetic architecture of normal variation in human pigmentation: An evolutionary perspective and model. Hum Mol Genet.

[pbio-0050171-b043] Akey JM, Swanson WJ, Madeoy J, Eberle M, Shriver MD (2006). TRPV6 exhibits unusual patterns of polymorphism and divergence in worldwide populations. Hum Mol Genet.

[pbio-0050171-b044] Stajich JE, Hahn MW (2005). Disentangling the effects of demography and selection in human history. Mol Biol Evol.

[pbio-0050171-b045] Rockman MV, Hahn MW, Soranzo N, Goldstein DB, Wray GA (2003). Positive selection on a human-specific transcription factor binding site regulating IL4 expression. Curr Biol.

[pbio-0050171-b046] Rockman MV, Hahn MW, Soranzo N, Zimprich F, Goldstein DB (2005). Ancient and recent positive selection transformed opioid cis-regulation in humans. PLoS Biol.

[pbio-0050171-b047] Stefansson H, Helgason A, Thorleifsson G, Steinthorsdottir V, Masson G (2005). A common inversion under selection in Europeans. Nat Genet.

[pbio-0050171-b048] Clark AG, Hubisz MJ, Bustamante CD, Williamson SH, Nielsen R (2005). Ascertainment bias in studies of human genome-wide polymorphism. Genome Res.

[pbio-0050171-b049] Kong A, Gudbjartsson DF, Sainz J, Jonsdottir GM, Gudjonsson SA (2002). A high-resolution recombination map of the human genome. Nat Genet.

[pbio-0050171-b050] Hudson RR (2002). Generating samples under a Wright-Fisher neutral model of genetic variation. Bioinformatics.

[pbio-0050171-b051] Prufer K, Muetzel B, Do HH, Weiss G, Khaitovich P (2007). FUNC: A package for detecting significant associations between gene sets and ontological annotations. BMC Bioinformatics.

