# Supplementary material for: A New Approach for Using Genome Scans to Detect Recent Positive Selection in the Human Genome
Source: PLoS Biol. 2007 Jun 19;5(7):e171. doi: 10.1371/journal.pbio.0050171 (PMC1892573; doi:10.1371/journal.pbio.0050171)
Supplement: Supplementary file 1 [file pbio.0050171.st001.doc]

Table S1: Proportions of Candidate Region Lists That Are Significant in the LRH test.

Pmn, P95 and P75 are related LRH tests use the mean, number of SNPs above 95%, or 75% as the test statistics respectively (see methods)
